# A Post–NGP *Mitsuokella jalaludinii* as a Therapeutic Candidate for Gout

**DOI:** 10.1002/mbo3.70410

**Published:** 2026-09-15

**Authors:** Mohammed Solayman Hossain, Sukyung Kim, Md Tareque Aziz, Izaz Ahmed, Md Sarower Hossen Shuvo, Hyewon Yang, Yunjeong Jang, Minseo Kim, Seohyeon Jang, Yusol Kim, Soeun Oh, Yunseo Nam, Hoonhee Seo, Ho‐Yeon Song

**Affiliations:** ^1^ Department of Microbiology and Immunology, School of Medicine Soonchunhyang University Cheonan Chungnam Republic of Korea; ^2^ K‐Microbiome Institute Asan Chungnam Republic of Korea; ^3^ Next‐Generation Microbiome Training Center Soonchunhyang University Asan Chungnam Republic of Korea

**Keywords:** gout, gut microbiome, hyperuricemia, *M. jalaludinii* PMC73, next‐generation probiotics (NGP), post–NGP

## Abstract

Gout is a common inflammatory disease closely associated with hyperuricemia, and its global prevalence is increasing, highlighting the need for safer and more effective therapeutic strategies. Recently, microbiome‐based therapeutics have emerged as promising alternatives, and the development of functionally validated NGP and their advanced forms, post–NGP strains, is gaining increasing attention. In this study, microbiome‐derived bacterial strains were screened based on their in vitro uric acid (UA)‐reducing capacity and hypoxanthine utilization. Among the tested strains, *Mitsuokella jalaludinii* exhibited the most pronounced uric acid reduction and growth enhancement in response to purine substrates compared to conventional lactic acid bacteria. This strain reduced uric acid levels by approximately 30% after 48 h (*p* < 0.001) and showed significantly increased growth in purine‐enriched minimal medium (*p* < 0.01). WGS further confirmed its strain‐level novelty, leading to its designation as PMC73, and revealed the presence of genetic features associated with purine metabolism and UA regulation. In a MSU‐induced RAW 264.7 macrophage model, PMC73 significantly reduced UA levels (*p* < 0.001) and modulated immune responses by increasing anti‐inflammatory cytokines while decreasing pro‐inflammatory cytokines. These effects were associated with suppression of the NLRP3–caspase‐1, along with downregulation of URAT1 and XOD, indicating coordinated regulation of uric acid production, reabsorption, and inflammatory pathways. Based on safety evaluation, including d‐lactate production, cytotoxicity assays and oral toxicity assessment in a mouse model, demonstrated that PMC73 exhibited no pathogenic or toxic properties. Therefore, PMC73 represents a promising microbiome‐based candidate with relevance to gout‐associated hyperuricemia, warranting further in vivo and clinical investigations.

## Graphical abstract

Schematic summary of the therapeutic mechanisms and evaluation of the post–NGP strain *M. jalaludinii* PMC73 for gout. PMC73 demonstrated uric acid–lowering efficacy through purine catabolism and hypoxanthine utilization, along with intracellular uric acid reduction in macrophages. Mechanistically, PMC73 suppressed URAT1 and XOD expression, leading to decreased uric acid production and reabsorption. In parallel, PMC73 inhibited NLRP3 inflammasome activation and caspase‐1 expression, resulting in reduced pro‐inflammatory cytokine (IFN‐γ) levels and increased anti‐inflammatory cytokines (IL‐4 and IL‐10). Comprehensive safety evaluation confirmed a microbiome‐safe profile with no cytotoxicity, virulence factors, or in vivo toxicity. These findings support PMC73 as a multi‐target microbiome therapeutic candidate for gout.

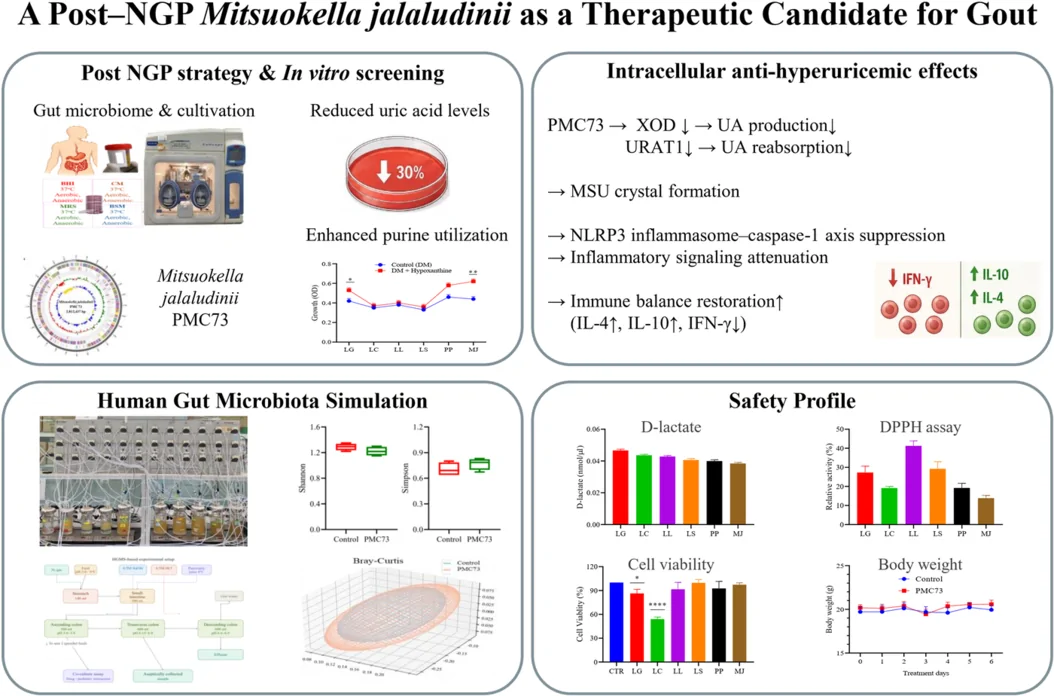

## 1 Introduction

Gout is the most prevalent form of inflammatory arthritis worldwide, affecting an estimated 41.2 million individuals globally, with its incidence continuing to rise across both developed and developing nation (Safiri et al. 2020). Epidemiological data indicate that gout prevalence has more than doubled over the past two decades (Dehlin et al. 2020), driven by shifting dietary patterns, increasing rates of obesity, metabolic syndrome, chronic kidney disease, and widespread use of uricosuric‐altering medications such as diuretics and low‐dose aspirin (Asghari et al. 2024). This escalating burden renders gout a significant public health concern with substantial implications for patient quality of life, healthcare expenditure, and comorbid disease management.

The pathophysiology of gout is predicated on hyperuricemia, characterized by serum uric acid (UA) levels surpassing the physiological saturation threshold of approximately 6.8 mg/dL (Narang and Dalbeth 2020). Uric acid, the end product of purine catabolism, is produced via the xanthine oxidoreductase (XOD)‐mediated transformation of hypoxanthine to xanthine and subsequently to uric acid (Furuhashi 2020). When serum urate surpasses its physiological saturation threshold, monosodium urate (MSU) crystals precipitate and accumulate in synovial joints and periarticular tissues, initiating the inflammatory cascade typical of gout. Current pharmacological management employs two primary strategies: acute flare resolution utilizing colchicine, NSAIDs, or corticosteroids, and long‐term urate‐lowering therapy (ULT) based on xanthine oxidase inhibitors (FitzGerald et al. 2020), primarily allopurinol and febuxostat. However, each agent carries a clinically significant adverse effect—allopurinol is associated with severe cutaneous hypersensitivity reactions including allopurinol hypersensitivity syndrome (AHS) and Stevens–Johnson syndrome, with disproportionate risk in HLA‐B*58:01 carriers prevalent in Asian populations (Sukasem et al. 2016); febuxostat has attracted regulatory scrutiny over cardiovascular safety concerns; and colchicine is limited by gastrointestinal toxicity and a narrow therapeutic index (White et al. 2018). These limitations collectively underscore the compelling necessity for novel, mechanistically distinct, and therapeutically safe alternatives for gout management.

Emerging evidence has made the gut microbiome as a potential determinant of purine metabolism and uric acid homeostasis (Méndez‐Salazar and Martínez‐Nava 2022; Tong et al. 2022), with the human intestine accounting for approximately one‐third of total daily extra‐renal uric acid excretion through uricolytic commensal communities (Yin et al. 2022). Gut dysbiosis in hyperuricemic and gouty individuals is characterized by depletion of uricolytic taxa and enrichment of purine‐generating microorganisms, while concurrent disruption of microbial immunomodulatory functions exacerbates the pro‐inflammatory cytokine imbalance central to gout pathogenesis (Lv et al. 2023). Next‐generation probiotics (NGPs)—distinguished from conventional *Lactobacillus* and *Bifidobacterium*‐based strains by their human gut commensal origin, mechanistic specificity, and disease‐targeted functional selection—represent a promising therapeutic framework to address this dual metabolic and inflammatory dysfunction (Abouelela and Helmy 2024). While the post–NGP concept shares conceptual overlap with postbiotics, it is proposed here as a functional extension of the NGP framework, emphasizing strain discovery pipelines that integrate culturomics, genomic validation, and disease‐specific mechanistic screening (Salminen et al. 2021). Prominent NGP candidates including *Akkermansia muciniphila* (L. Zhang et al. 2022) and *Faecalibacterium prausnitzii* (Shao et al. 2017) have demonstrated efficacy across metabolic and inflammatory conditions, and strains possessing intrinsic uricolytic or purine‐catabolizing capacity offer a physiologically integrated and pharmacologically safer alternative to conventional ULT for hyperuricemia management.

Probiotics are conventionally defined as live microorganisms that confer a measurable health benefit when administered in adequate amounts, per the FAO/WHO framework. Despite this broad definition, approved strains remain concentrated in a few genera—chiefly Lactobacillus and Bifidobacterium—valued for safety history over functional range (Abouelela and Helmy 2024). Next‐generation probiotics (NGPs) arose to broaden this pool, using metagenomic sequencing and functional screening to identify disease‐relevant, often previously uncultured gut commensals directly from the microbiome, rather than relying on traditional fermentation strains (Kern et al. 2026). This expanded candidate diversity but introduced new challenges: many NGPs are strict anaerobes with poor oxygen tolerance and viability through manufacturing and gastrointestinal transit is still difficult to preserve (Lalowski and Zielińska 2024). In this context, we introduce the concept of post–NGP. Rather than simply denoting taxonomically new organisms, post–NGP describes a systematic framework for uncovering microbiome‐derived therapeutic candidates across the full breadth of gut microbial diversity. It moves past empirical, narrowly focused strain selection by combining culturomics‐based isolation, genomic profiling, function‐driven screening, and physiological validation into a single discovery pipeline. This layered approach allows for a more structured exploration of microbial diversity within the gut's ecological complexity, helping surface therapeutically promising candidates that earlier strategies overlooked.

Even though these gut‐derived NGP candidates have therapeutic potential, the systematic isolation, thorough functional characterization, and mechanistic validation of those with specific anti‐hyperuricemic activity are still not well‐studied. This study presents the isolation of bacterial strains and their methodical evaluation for anti‐hyperuricemic activity via uric acid and hypoxanthine utilization assays. A primary candidate, *M. jalaludinii* PMC73, was selected for extensive probiotic characterization and safety evaluation. After that, its mechanistic anti‐gout effectiveness was tested in a RAW 264.7 macrophage cell model that was triggered by MSU crystals. The focus was on modulating uric acid, reducing ROS, suppressing the NLRP3 inflammasome, and rebalancing cytokines. The findings presented herein establish PMC73 as a novel, safe, and mechanistically validated post–NGP candidate with significant therapeutic potential for gout and hyperuricemia management, providing a strong preclinical foundation for future in vivo and clinical investigation.

## 2 Material and Method

### 2.1 Isolation of NGP from Clinical Samples

The experiment was conducted to isolate an obligate commensal bacterium with potential as an NGP, expected to surpass existing NGP strains in probiotic efficacy. Accordingly, stool sample collection from healthy individuals at Soonchunhyang University Seoul Hospital. Using culturomics techniques, we optimized media compositions, temperature, and oxygen requirements to isolate the target strains. Samples underwent serial dilution from 1 g in 1 mL PBS through 10^−1^ to 10^−6^, then streaked onto MRS (de Man, Rogosa and Sharpe), CM (Chopped Meat), BHI (Brain Heart Infusion*)* and BSM (Bifidobacterium Selective Media) agar plates (Kisanbio, Korea) and incubated anaerobically (Baker Ruskin, Canada) at 37°C for 48 h. After colony development, isolates were transferred into respective broths under identical conditions and stored at −80°C in 30% glycerol for further use.

### 2.2 16S rRNA‐Based Identification of Candidate Strains

Cultured strains were sent to a commercial sequencing company (Biofact, Korea) for 16S rRNA gene‐based identification. Briefly, genomic DNA was extracted and PCR amplification was carried out using primers 27F (5′‐AGAGTTTGATCCTGGCTCAG‐3′) and 1492R (5′‐GGTTACCTTGTTACGACTT‐3′) on a Hushrun PCR cycler (Biofact, Korea) (Shuvo et al. 2025). The resulting PCR products were purified and sequenced using an ABI PRISM 3730XL DNA analyzer (Applied Biosystems, USA) with a BigDye Terminator v3.1 Cycle Sequencing Kit (Thermo Fisher Scientific, USA). Generated sequences were then compared against the NCBI GenBank database using BLAST for strain‐level identification.

### 2.3 Species‐Specific PCR Identification

Genomic DNA was extracted from cell pellets using the QIAamp DNA Microbiome Kit (Qiagen, Germany) according to the manufacturer's instructions. Species‐specific primers targeting *L. graminis*, *P. pentosaceus*, *L. lactis*, *L. sakei*, *L. curvatus*, and *M. jalaludinii* were used for molecular identification (Table 1). PCR amplification was performed using Maxime PCR Premix (iNtRON Biotechnology, Korea) on a 96‐Well Thermal Cycler (Thermo Fisher Scientific, USA). Amplicons were resolved by electrophoresis on a 1% (w/v) agarose gel and visualized under UV illumination using a ChemiDoc Imaging System (Bio‐Rad, USA). Additionally, virulence gene screening of strain PMC73 was carried out using gene‐specific primers with the same PCR apparatus as previously described (Barman et al. 2025).

### 2.4 Whole‐Genome Sequencing of Probiotic Strain

WGS was performed to characterize the genome of the candidate strain. Strain PMC73, obtained from a human fecal sample in anaerobic conditions, was subjected to extensive genomic characterization, commencing with initial 16S rRNA gene identification through Biofact (Korea) and NCBI BLAST analysis. CJ Bioscience (Korea) sequenced genomic DNA using the PacBio RSII platform and put it together from scratch into a complete circular chromosome for high‐resolution classification. The EzBioCloud pipeline and the Clusters of Orthologous Groups (COG) database were then used to annotate the genome and put it into functional groups to find CDSs, rRNAs, and tRNAs. Lastly, the taxonomic status of PMC73 was determined by computing the Orthologous Average Nucleotide Identity (OrthoANI) in relation to reference *Mitsuokella* species and creating a phylogenomic tree, utilizing a species delineation threshold of 95%–96% ANI.

### 2.5 Cell Line and Culture Conditions

The murine macrophage RAW 264.7 cell line (RRID: CVCL_0493), derived from male peritoneal macrophages, was procured from the Korean Cell Line Bank (KCLB, Korea) in 2021. Cells were maintained in DMEM (Gibco, USA) supplemented with 10% fetal bovine serum (FBS; Gibco, USA) and 1% antibiotic solution (100 U/mL penicillin; HyClone, USA) at 37°C in a humidified 5% CO_2_ atmosphere. Prior to experimental use, the cell line was authenticated by short tandem repeat (STR) profiling, yielding a ≥ 95% match to the reference profile, and confirmed free of mycoplasma contamination using a PCR‐based detection assay. RAW 264.7 is not listed among misidentified or contaminated cell lines in the ICLAC database.

### 2.6 Uric Acid Utilization Assay

The uric acid utilization capacity of NGP strains was evaluated using a colorimetric assay (Wang et al. 2025). NGP strains were cultured overnight at 37°C, harvested by centrifugation, washed with sterile PBS (pH 7.4), and resuspended to 10^8^ CFU/mL in fresh broth supplemented with 80 mg/L uric acid (Sigma‐Aldrich, USA). Cell pellets were dispensed into tubes and incubated anaerobically at 37°C for 48 h. Following incubation, supernatants were collected after centrifugation, and 25 μL was mixed with 25 μL assay reagent from the Uric Acid Colorimetric Assay Kit (Elabscience, China) along with the protein precipitant. Absorbance was measured at 690 nm using a microplate reader (Perkin‐Elmer, USA) against a standard curve. All assays were performed in triplicate and results expressed as mean ± SD. Uric acid content was calculated as: UA content (mg/L) = (*A*_690 _− blank)/slope.

### 2.7 Purine Utilization Assay

The purine utilization ability of NGP strains was assessed by monitoring growth in hypoxanthine‐supplemented defined medium, following the protocol previously described by Cheng et al. (Lee et al. 2022). A chemically defined medium (CDM) was prepared according to the referenced CDM formulation and pre‐warmed to 37°C. Hypoxanthine was dissolved using sequential acid–base adjustment and filter‐sterilized to prepare a purine stock solution, which was added to DM at a final concentration of 400 μM. Bacterial suspensions adjusted to 10^8^ CFU/mL were inoculated at 1% (v/v) into both hypoxanthine‐supplemented DM and unsupplemented DM as control. Cultures were incubated anaerobically at 37°C for up to 8 h, with OD_600_ measured at defined time points using a spectrophotometer. Cell growth was expressed relative to the control, with the initial media OD set as 1.0. The experiment was performed in three independent replicates and results are presented as mean ± SD.

### 2.8 Uric Acid Quantification

The effect of PMC73 on uric acid levels was evaluated in MSU‐induced inflammatory RAW 264.7 macrophages. Cells (1 × 10^5^ per mL) were cultured in DMEM supplemented with 10% FBS and 1% penicillin at 37°C under 5% CO_2_ for 24 h. Cells were then washed twice with PBS and exposed to MSU crystals (200 μg/mL) in serum‐free DMEM for 2 h to induce gout‐like inflammation (Pan et al. 2021). After stimulation, residual MSU crystals were removed by PBS washing, and the following treatments were applied in serum‐free DMEM: non‐treated control, MSU‐only group, PMC73 group (8% v/v of a 1 × 10^8^ CFU/mL bacterial extract), and colchicine group (1 μM) (Orlowsky et al. 2014). All groups were incubated for 48 h at 37°C under 5% CO_2_. Uric acid levels in the conditioned media were measured using the Uric Acid Kit (Elabscience, China) per the manufacturer's protocol. Absorbance was measured at 690 nm using a microplate reader, and concentrations were determined from a standard curve prepared in parallel.

### 2.9 Reactive Oxygen Species Measurement

For ROS detection, cells were cultured under the same conditions described for the uric acid assay. Briefly, cells were stimulated with MSU crystals, treated with PMC73, and then incubated for an additional 6 h. Following incubation, cells were washed once with HBSS and loaded with the ROS detection probe (Promega, USA) according to the manufacturer's protocol. Fluorescence was measured using a microplate reader at an excitation wavelength of 495 nm. ROS levels were expressed as relative fluorescence units (RFU), normalized to cell number or viability, and compared across all experimental groups.

### 2.10 XOD Analysis Assay

For XOD analysis, the Raw cells were seeded in cell culture flask followed by treated with probiotic and Allopurinol as per previously described protocol (Na et al. 2021). Cells were initially treated with probiotic and drug for better effect then induced gout by MSU. After 24 h of probiotic treatment, sample was centrifuged at 3000 rpm for 10 min, and the supernatant was collected analyzed following Elabscience XOD kit, China. A standard curve was prepared along with the sample in all cases. Colorimetric assay reagent was added, mixed, and kept at room temperature for 15 min. Samples were dispensed in a 96‐well plate to measure the OD values of each well at 690 nm and 550 nm using a microplate reader at 30‐min intervals.

### 2.11 Cytokine and Gene Expression Analysis

To assess the inflammatory response at both the protein and transcriptional levels, cytokine concentrations and gene expression profiles were analyzed from the same treated cell cultures. Cells were stimulated with MSU crystals, treated with PMC73, and incubated for an additional 6 h before sample collection. For cytokine quantification, cell culture supernatants were carefully collected and centrifuged at 4,000 rpm for 10 min to remove cellular debris. The clarified supernatants were transferred into fresh microcentrifuge tubes and stored appropriately until analysis. Concentrations of IL‐4, IL‐10 and IFN‐γ were determined using the LEGEND MAX Mouse ELISA Kit (BioLegend, USA) in accordance with the manufacturer's instructions. Absorbance was recorded using a microplate reader, and cytokine concentrations were extrapolated from standard calibration curves.

For gene expression analysis, supernatants were aspirated and adherent cells were scraped and collected. Total RNA was extracted using the RNeasy Mini Kit (Qiagen, Germany) following the manufacturer's protocol. Purified RNA was reverse‐transcribed into cDNA using the iScript cDNA Synthesis Kit (Bio‐Rad, USA). Relative mRNA expression levels of NLRP3, caspase‐1, URAT1, and XOD (Table 2) were subsequently quantified by RT‐PCR on a CFX96 Real‐Time PCR Detection System (Bio‐Rad, USA). Expression levels were calculated using the 2^−ΔΔCT^ method, normalized to β‐actin as the housekeeping reference gene (Hossain et al. 2025).

### 2.12 Assessment of d‐Lactate Production

d‐lactate production was evaluated as a key safety parameter, given its association with d‐lactic acidosis (Hossain et al. 2024). Candidate strains were cultured in broth at 37°C for 24 h, after which supernatants were collected and d‐lactate concentrations measured using the d‐Lactate Assay Kit (Abcam, USA) per the manufacturer's protocol. A standard d‐lactate solution was used as a reference, and absorbance was measured with a microplate reader.

### 2.13 Free Radical Scavenging Activity

DPPH radical scavenging activity was used to assess the antioxidant potential of NGP strains (Barman et al. 2025). Strains were cultured overnight in broth at 37°C, centrifuged, and cell‐free supernatants (CFS) were collected and filtered through a 0.22 μm syringe filter. Subsequently, 1 mL of DPPH solution (0.05 mM) was mixed with 0.1 mL of CFS, vortexed, and incubated in the dark at room temperature for 30 min. Absorbance was measured at 517 nm using a spectrophotometer. l‐Ascorbic acid (50 μM) and PBS served as positive and negative controls, respectively. Scavenging activity was calculated as: DPPH scavenging activity (%) = [1 − (*A*_s_ − *A*_b_)/*A*_CTRL_] × 100.

### 2.14 Cytotoxicity Assay

Cell viability was evaluated to confirm the safety of probiotic extracts using two mitochondrial activity‐based assays. For the WST assay, RAW 264.7 cells (1 × 10^5^ cells/mL) were seeded in a 96‐well plate using DMEM (Gibco, USA) and incubated overnight at 37°C with 5% CO_2_, then washed with PBS. EZ‐Cytox reagent (Dogen, Korea) was added (20 µL/well), incubated for 30 min, briefly shaken for 1 min, and absorbance was read at 450 nm using a microplate reader.

### 2.15 In Vivo Oral Toxicity Assessment

Male C57BL/6J mice (5 weeks old) were procured from Doo Yeol Biotech (Korea) and maintained under standard laboratory conditions (20°C–25°C, 30%–70% relative humidity, 12‐h light/dark cycle) with ad libitum access to tap water and a regular diet. Following 1 week of acclimatization, oral toxicity was assessed by administering probiotics once daily, 5 days per week. Animals were divided into two groups (6/group): one receiving probiotics cultured in CM Broth (1 × 10^8^ CFU/mL) and the other receiving sterile saline as a control. Throughout the treatment period, all animals were monitored for clinical signs, mortality, and body weight changes.

### 2.16 Metagenomic Analysis of Microbial Strain‐Induced Dysbiosis

To investigate gut dysbiosis, the human gut microbiome was modeled using the Human Gut Microbiome Simulation (HGMS) system (Figure 7A), adapted from the SHIME platform (ProDigest, Belgium) (Shuvo et al. 2025). This multi‐compartment in vitro system replicates gastrointestinal physiology and was inoculated with a 5% (w/v) fecal suspension prepared from freshly collected stool of a healthy adult donor (antibiotic‐free for ≥ 3 months), processed under strict anaerobic conditions. Continuous mixing at 300 rpm ensured microbial homogeneity throughout. Two parallel HGMS units were operated simultaneously—one as an untreated control and one receiving daily administration of 10 mL PMC73 at a defined concentration (CFU/mL).

Metagenomic analysis was conducted following previously established protocols. Total genomic DNA was extracted from collected samples using the QIAamp DNA Mini Kit (Qiagen, Hilden, Germany) and quantified using a Qubit 4 Fluorometer (Thermo Fisher Scientific, Waltham, MA, USA). The V4 hypervariable region of the 16S rRNA gene was amplified using specific primers and PCR with KAPA HiFi HotStart ReadyMix (Kapa Biosystems, Wilmington, MA, USA). Amplicons were purified using AMPure XP beads (Beckman Coulter, Brea, CA, USA). Sequencing libraries were prepared using the Nextera XT DNA Library Prep Kit and sequenced on the Illumina iSeq. 100 (Illumina, San Diego, CA, USA). Raw sequence data were processed and analyzed using QIIME (version 1.9.1) for taxonomic classification and diversity analysis (Bolyen et al. 2019).

### 2.17 Statistical Analysis

All data are presented as the mean and standard deviation (SD) for in vitro and cellular assays (*n* = 3 or *n* = 6 independent biological replicates), or as the mean and standard error of the mean (SEM) for in vivo animal experiments, as indicated in the respective figure legends. Genomic analysis, Average Nucleotide Identity (ANI) calculation, and phylogenomic tree construction—were performed using CL Genomics software (ChunLab, Seoul, South Korea) according to standard parameter thresholds (96% OrthoANI cut‐off for species demarcation).

Statistical differences among multiple treatment groups were determined using a one‐way analysis of variance (ANOVA) followed by Tukey's post hoc test for multiple comparisons. For pairwise comparisons of microbial alpha (Shannon diversity index) and beta (Bray–Curtis dissimilarity) metrics in the ex vivo simulator, a paired Student's *t*‐test was applied. Homogeneity of variance and normal distribution were verified prior to parametric testing. All statistical analyses and graphical renderings were performed using GraphPad Prism. A *p* < 0.05 was considered statistically significant.

## 3 Results

### 3.1 Isolation and Characterization NGP Strains

**Figure 1:**
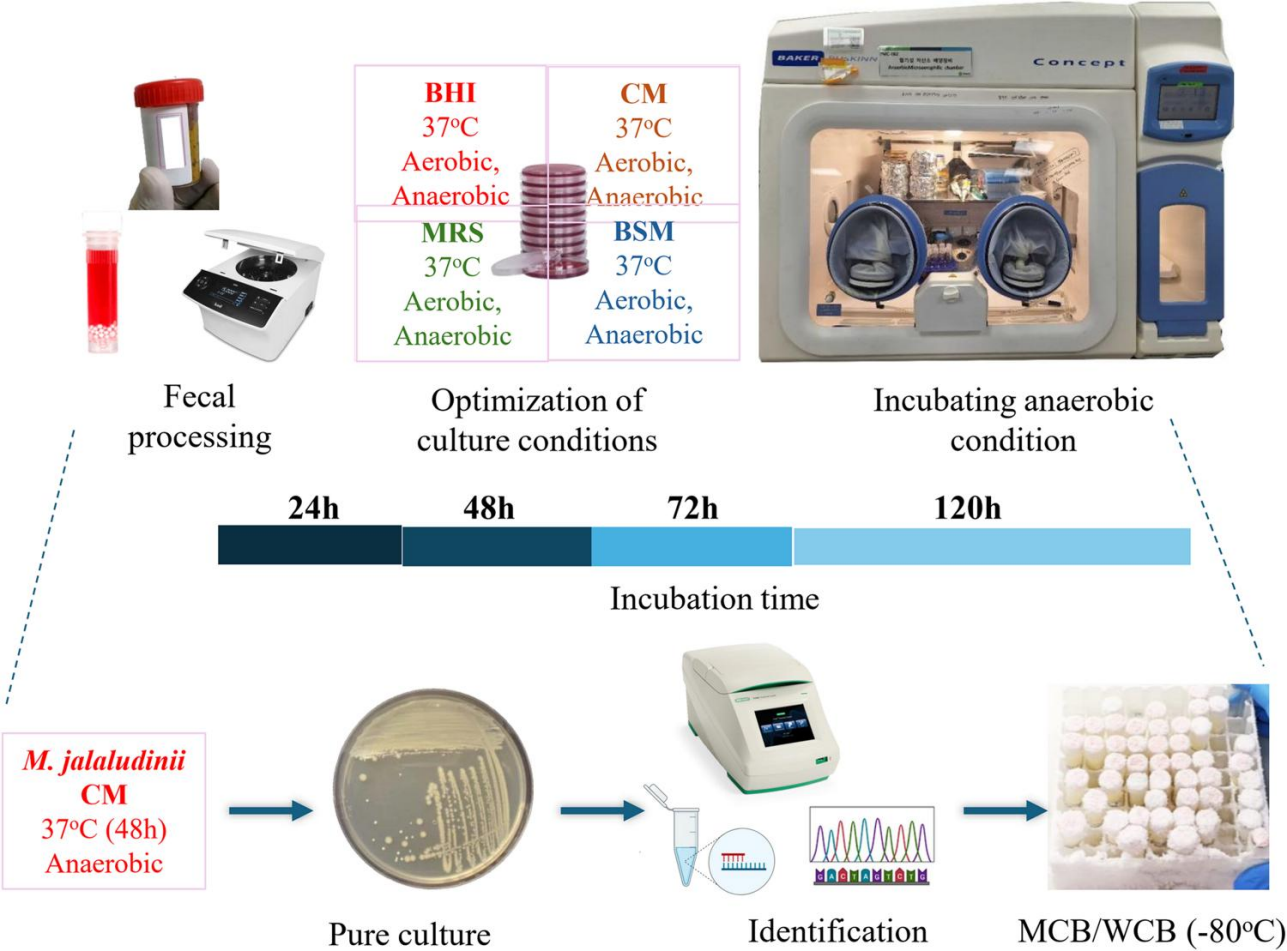
Isolation and identification process of NGPs from clinical stool samples using culturomics‐based approaches. Stool samples were collected, homogenized, and centrifuged to prepare for inoculation. Culture conditions were systematically optimized across four media – BHI, CM, MRS, BSM—under both aerobic and anaerobic conditions at 37°C, with incubation periods extending from 24 to 120 h. Colonies grown on CM agar under anaerobic conditions at 37°C for 48 h were selected for downstream processing. Pure cultures were subjected to DNA extraction, 16S rRNA gene sequencing, and NCBI BLAST‐based taxonomic identification, confirming the isolate as *M. jalaludinii* (PMC73). Confirmed strains were preserved as bacterial stocks at −80°C for long‐term storage.

A flowchart summarizing the culturomics‐based novel strain development strategy is presented in Figure 1. The workflow commenced with the collection, enrichment, and anaerobic culturing of fecal samples in an anaerobic chamber, which facilitated the development of primary cultures. Microbial colonies were then isolated, and single colonies were picked to establish pure cultures. The taxonomic analysis of the isolated NGP strains was conducted through 16S rRNA gene sequencing. The results confirmed that all isolates showed a high level of sequence homology (98%–99%) with known lactic acid bacterial species, indicating accurate species‐level identification (Table 3). All isolates showed high sequence similarity to known lactic acid bacterial species, including *M. jalaludinii, Latilactobacillus sakei, Lactococcus lactis, Pediococcus pentosaceus, and Latilactobacillus curvatus*. These findings confirm that the isolates belong to diverse genera commonly associated with fermented foods and probiotic functions, including *Lactiplantibacillus, Latilactobacillus, Lactococcus, Mitsuokella, and Pediococcus*, supporting their potential relevance for subsequent probiotic characterization. Species‐specific PCR amplification successfully confirmed the identities of the candidate probiotic strains, with distinct amplicons observed for each target species on 1% agarose gels (Table 1). All strains produced expected band sizes corresponding to their respective primer pairs, verifying the 16S rRNA sequencing results. These results provide orthogonal molecular confirmation of the isolated strains' taxonomic assignments, supporting their identification for downstream probiotic functional assays.

**Table 1:** Species‐specific PCR primers used for molecular identification of probiotic bacterial strains.

Bacterial species	Primers	Primer sequence	Identification result
*L. graminis*	graminis_F	GGT GGT GCG ATT ACT GGA CT	+
	graminis_R	CCA TTA CTG AGC CGG TTA GA	
*P. pentosaceus*	acidi_F	TTA GCG GCC CTT TGC GGT TC	+
	acidi_R	ATT GCC GCG AAG AAG CCC AC	
*L. lactis*	LcLspp‐F	GTTGTATTAGCTAGTTGGTGAGGTAAA	+
	LacreR	GGGATCATCTTTGAGTGAT	
*L. sakei*	sakei_F	AGG CGC TTC AAT GTT ATC GG	+
	sakei_R	TCG CTG GTT GCT TGA TGC TA	
*L. curvatus*	curvatus_F	TCC AAG GAA GAA CGC TGT CA	+
	curvatus_R	AAG ACC GAA TTG CGC GAG TT	
*M. jalaudinii*	cel5C‐F	TCAAGGACTGCGAGGACTGG	+
	cel5C‐R	ACGGGAGTCGCTCTTGTGA	

**Table 2:** List of primers that have been used for determining gene expression of MSU treated cells.

Primers	Forward sequence	Reverse sequence
NLRP3	5′‐GTGGTGACCCTCTGTGAGGT‐3′	5′‐TCTTCCTGGAGCGCTTCTAA‐3′
Caspase1	5′‐TATCCAGGAGGGAATATGTG‐3′	5′‐ACAACACCACTCCTTGTTTC‐3′
URAT1	5′‐CGTGGGACCTGGTATGTAACT‐3′	5′‐CCAAACCTATCTGAGGCATGG‐3′
XOD	5′‐ATGACGAGGACAACGGTAGAT‐3′	5′‐TCATACTTGGAGATCATCACGGT‐3′
ß‐actin	5′‐GGTCATCACTATTGGCAACG‐3′	5′‐ACGGATGTCAACGTCACACT‐3′

**Table 3:** Identification of NGP strains based on 16S rRNA gene sequencing.

ID	NCBI Ref.	Organism	Scores (bits)	Identity
MJ	NR_028840.1	*Mitsuokella jalaludinii strain M9*	2678 (1450)	99%
	NR_027596.1	*Mitsuokella multacida strain DSM 20544*	2523 (1366)	99%
LS	NR_042443.1	*Latilactobacillus sakei subsp. sakei strain DSM 20017*	2767 (1498)	99%
	NR_113821.1	*Latilactobacillus sakei strain NBRC 15893*	2761 (1495)	99%
LL	NR_040955.1	*Lactococcus lactis strain NCDO 604*	2715 (1470)	99%
	NR_113960.1	*Lactococcus lactis strain NBRC 100933*	2706 (1465)	99%
PP	NR_042058.1	*Pediococcus pentosaceus strain DSM 20336*	2723 (1474)	99%
	NR_042401.1	*Pediococcus stilesii strain FAIR‐E 180*	2606 (1410)	98%
LC	NR_042437.1	*Latilactobacillus curvatus strain DSM 20019*	2724 (1455)	99%
	NR_113334.1	*Latilactobacillus curvatus strain NBRC 15884*	2719 (1472)	99%
LG	NR_114916.1	*Latilactobacillus graminis strain G90*	2684 (1453)	99%
	NR_113821.1	*Latilactobacillus sakei strain NBRC 15893*	2747 (1487)	99%

### 3.2 Anti‐Hyperuricemia Screening Result

**Figure 2:**
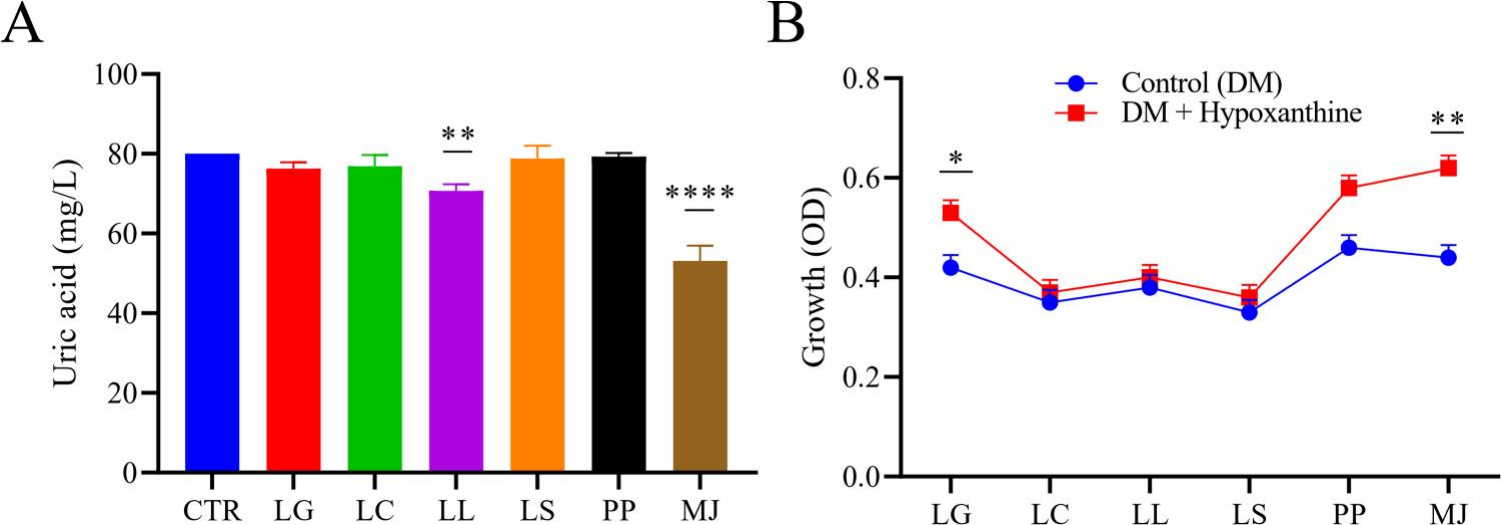
Screening of NGP strains for anti‐hyperuricemic activity based on uric acid degradation capacity and hypoxanthine assimilation ability. (A) Residual UA concentrations (mg/L) measured after 48 h of anaerobic incubation of six candidate strains (CTR: untreated control; LG: *L. graminis*; LC: *L. curvatus*; LL: *L. lactis*; LS: *L. sakei*; PP: *P. pentosacus*; MJ: *M. jalaludinii*) in uric acid‐supplemented medium. The untreated group served as the baseline control. Strain MJ demonstrated the most pronounced uric acid degradation, reducing residual levels to approximately 52 mg/L (*p* < 0.0001), while strain LL showed a moderate but statistically significant reduction (*p* < 0.01). (B) Growth of six probiotic strains measured by optical density (OD600) after 8 h of anaerobic incubation at 37°C in defined medium (DM) alone (blue line) or DM supplemented with 400 μM hypoxanthine (red line). Strains MJ and LG exhibited significantly enhanced growth upon hypoxanthine supplementation, with mean OD600 values reaching 0.62 and 0.53, respectively, compared to 0.44 and 0.42 in their unsupplemented controls (*p* < 0.01 and *p* < 0.05, respectively). Data are presented as mean ± SD. Statistical significance was determined by one‐way ANOVA followed by Tukey's post hoc test.

To identify LAB strains with potential anti‐hyperuricemic activity, uric acid degradation capacity and hypoxanthine assimilation ability were evaluated across 6 candidate strains (Figure 2). Among the six LAB strains tested, uric acid degradation varied considerably after 48 h of anaerobic incubation (Figure 2A). Strain MJ exhibited the most significant reduction in residual uric acid levels compared to the untreated control, achieving the lowest recorded value (~52 mg/L, *p* < 0.0001). Strain LL also showed a moderate but statistically significant reduction (*p* < 0.01). The remaining strains—LG, LC, LS, and PP—displayed minimal uric acid degradation, with residual levels comparable to the control group and no statistical significance. Growth of six LAB strains in DM medium supplemented with 400 μM hypoxanthine was assessed by OD600 after 8 h of anaerobic incubation at 37°C and compared to unsupplemented DM controls (Figure 2B). Hypoxanthine supplementation enhanced growth in a strain‐dependent manner. The most notable increases were observed in strains MJ and LG, which reached mean OD600 values of 0.62 and 0.53, compared to 0.44 and 0.42 in their respective controls, and these differences were statistically significant (*p* < 0.01 and *p* < 0.05, respectively). The remaining strains—LC, LL, LS, and PP—showed only marginal increases in OD600 that did not reach statistical significance. These results suggest that strains MJ and LG are promising anti‐gout probiotic candidates, as their ability to assimilate exogenous hypoxanthine actively may reduce its conversion to uric acid, thereby potentially alleviating hyperuricemia.

### 3.3 Whole‐Genome Analysis Result of PMC73

**Figure 3:**
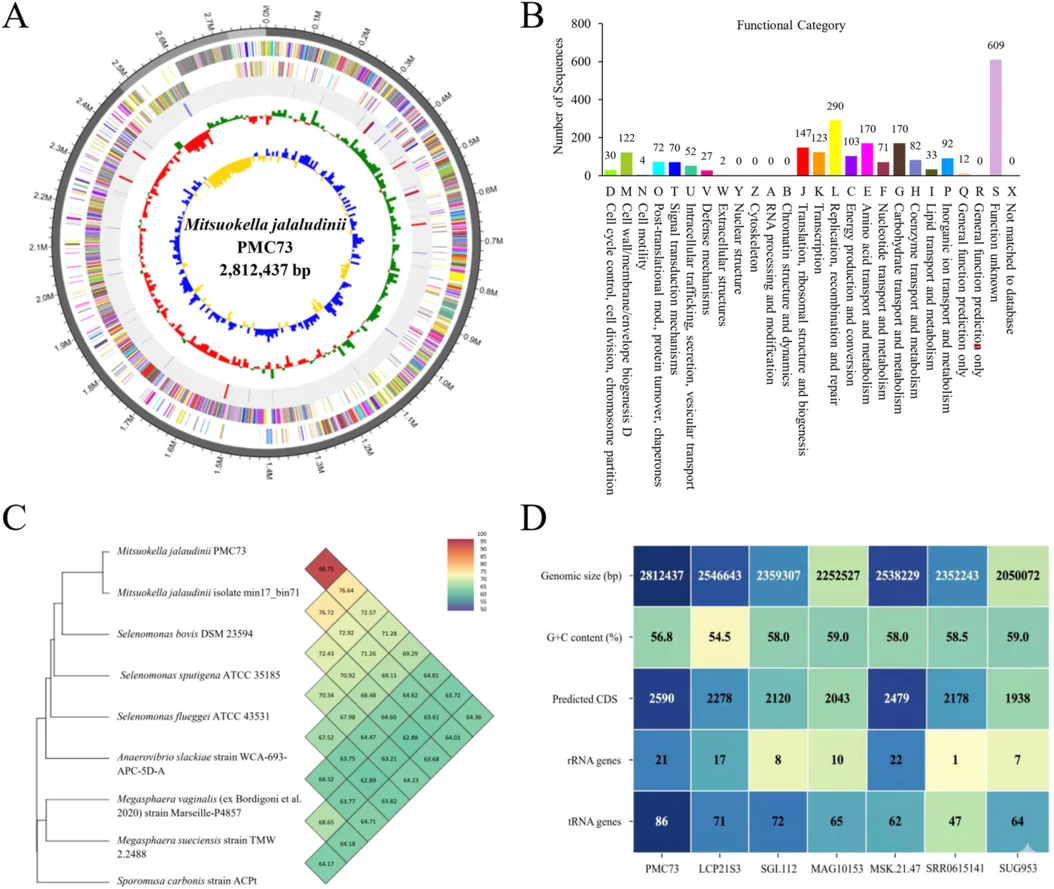
Whole‐genome characterization of *Mitsuokella jalaludinii* PMC73. (A) Circular genome map of *M. jalaludinii* PMC73 (2,812,437 bp). Moving from the outermost to the innermost rings, the map displays sense and antisense coding sequences color‐coded by COG functional category, followed by RNA gene tracks (tRNA in red; rRNA in blue), GC content (red, above average; green, below average), and GC skew (yellow, positive; blue, negative). The map was generated using CL Genomics. (B) Distribution of predicted genes across COG functional categories, reflecting the metabolic and cellular repertoire encoded within the PMC73 genome. (C) Phylogenomic tree and pairwise OrthoANI matrix comparing PMC73 with closely related strains. The 96% OrthoANI threshold conventionally used for species‐level demarcation is indicated; the ANI value between PMC73 and its nearest neighbor, *M. jalaludinii* isolate min17_bin71, was 96.77%, confirming conspecific status. (D) Comparative overview of core genomic features—including genome size, G + C content, predicted CDS count, and rRNA/tRNA gene complement—across representative *M. jalaludinii* strains.

Whole‐genome sequencing analysis was performed for the strain to enable its genomic characterization (Figure 3). The results of the whole‐genome sequencing analysis for the strain and identified as unique strain of *M. jalaludinii* PMC73. The genome consists of a single circular chromosome totaling 2,812,437 bp with a GC content of 56.8% (Figure 3A). Genomic annotation identified 2590 coding sequences (CDSs), 21 rRNA genes, and 86 tRNA genes, while functional classification via the COG database revealed a robust representation of genes dedicated to metabolism, cellular processes, and information storage (Figure 3B). Comparative genomic analysis using the OrthoANI approach showed that strain PMC73 shares the highest sequence similarity (96.77%) with *M. jalaludinii* isolate min17_bin71 (Figure 3C). Based on this high identity, which exceeds the species delineation threshold, the isolate was taxonomically confirmed as *M. jalaludinii*. However, further comparative analysis revealed distinct phenotypic characteristics and a unique isolation source compared to previously reported strains of this species (Figure 3D). Consequently, we identified this isolate as a novel strain of *M. jalaludinii PMC73*.

### 3.4 Uric Acid, ROS and XOD Quantification

**Figure 4:**
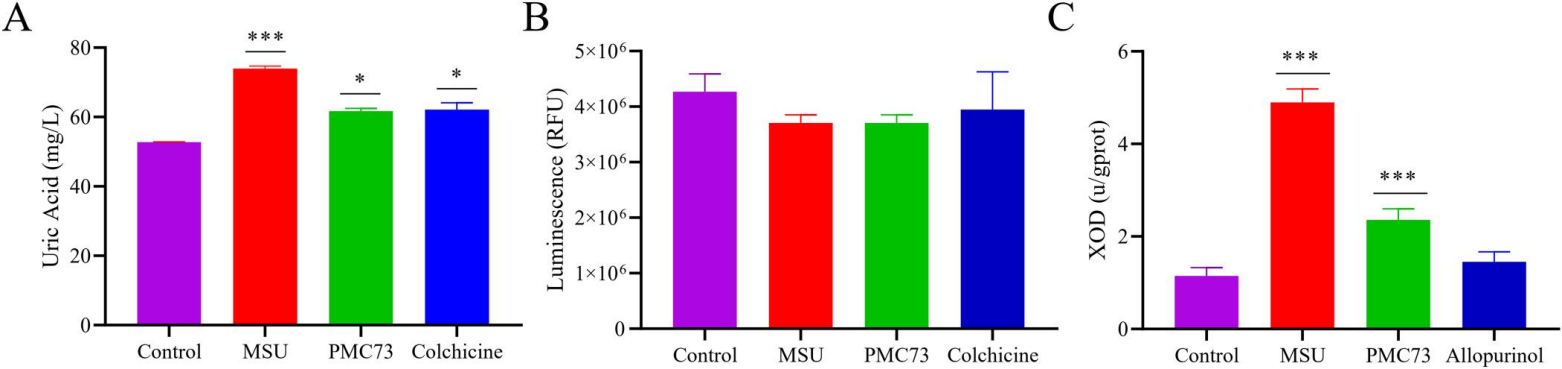
Uric acid levels, intracellular ROS, and xanthine oxidase (XOD) activity in MSU‐stimulated RAW 264.7 cells. (A) Measurement of extracellular uric acid concentrations (mg/L) in culture supernatants after 48 h treatments. MSU stimulation significantly increased uric acid versus control (*p* < 0.001); both PMC73 and colchicine markedly reduced levels versus MSU. (B) Intracellular ROS levels measured as relative luminescence units (RFU × 10^6^) using Promega ROS assay. No significant differences observed across groups (*p* > 0.05), suggesting PMC73 acts via ROS‐independent mechanisms. (C) Xanthine oxidase (XOD) activity (U/gprot) following treatments. MSU significantly elevated XOD activity compared to the control group (*p* < 0.001), whereas PMC73 treatment significantly attenuated this increase. Data represent mean ± SD (*n* = 3) (significance one‐way ANOVA with Tukey's post hoc test). Control: untreated; MSU: 200 μg/mL monosodium urate.

MSU stimulation significantly elevated uric acid levels in RAW 264.7 cells, which were effectively reduced by PMC73 CFS treatment, while ROS levels remained statistically unchanged (Figure 4). As illustrated in Figure 4A, MSU stimulation produced a marked and statistically significant elevation in uric acid levels compared to the untreated control group (*p* < 0.001), confirming successful induction of a gout‐like inflammatory state in macrophages. Treatment with PMC73 extract substantially reduced extracellular uric acid concentrations relative to the MSU‐only group (*p* < 0.05), bringing levels closer to those observed in the non‐treated control. A comparable reduction was also evident in the colchicine‐treated group (*p* < 0.05), a standard anti‐gout therapeutic used here as a positive control. MSU‐stimulated cells exhibited a modest reduction in luminescence (~ 3.6 × 10^6^ RFU) compared to the untreated control group (~ 4.2 × 10^6^ RFU), though this difference did not appear statistically significant given the overlapping error bars (Figure 4B). Treatment with PMC73 extract yielded luminescence values comparable to the MSU group, suggesting no substantial further modulation of ROS under these experimental conditions. The colchicine‐treated group displayed an intermediate level of luminescence, also within the range of the control. Furthermore, xanthine oxidase (XOD) activity was quantified to evaluate the potential enzymatic mechanisms involved (Figure 4C). MSU stimulation significantly elevated cellular XOD activity compared to the untreated control group (*p* < 0.001). However, treatment with PMC73 extract robustly attenuated this MSU‐induced increase in XOD activity (*p* < 0.001), demonstrating an efficacy comparable to the prominent XOD inhibitor allopurinol, which served as a positive control. Overall, the similar degree of uric acid reduction between PMC73 and Colchicine suggests that PMC73 exhibits uric acid‐lowering and XOD inhibiting activity in vitro that is on par with conventional pharmacological interventions, and the data replicates the result we observed in vitro screening.

### 3.5 Cytokine and Gene Expression Analysis

**Figure 5:**
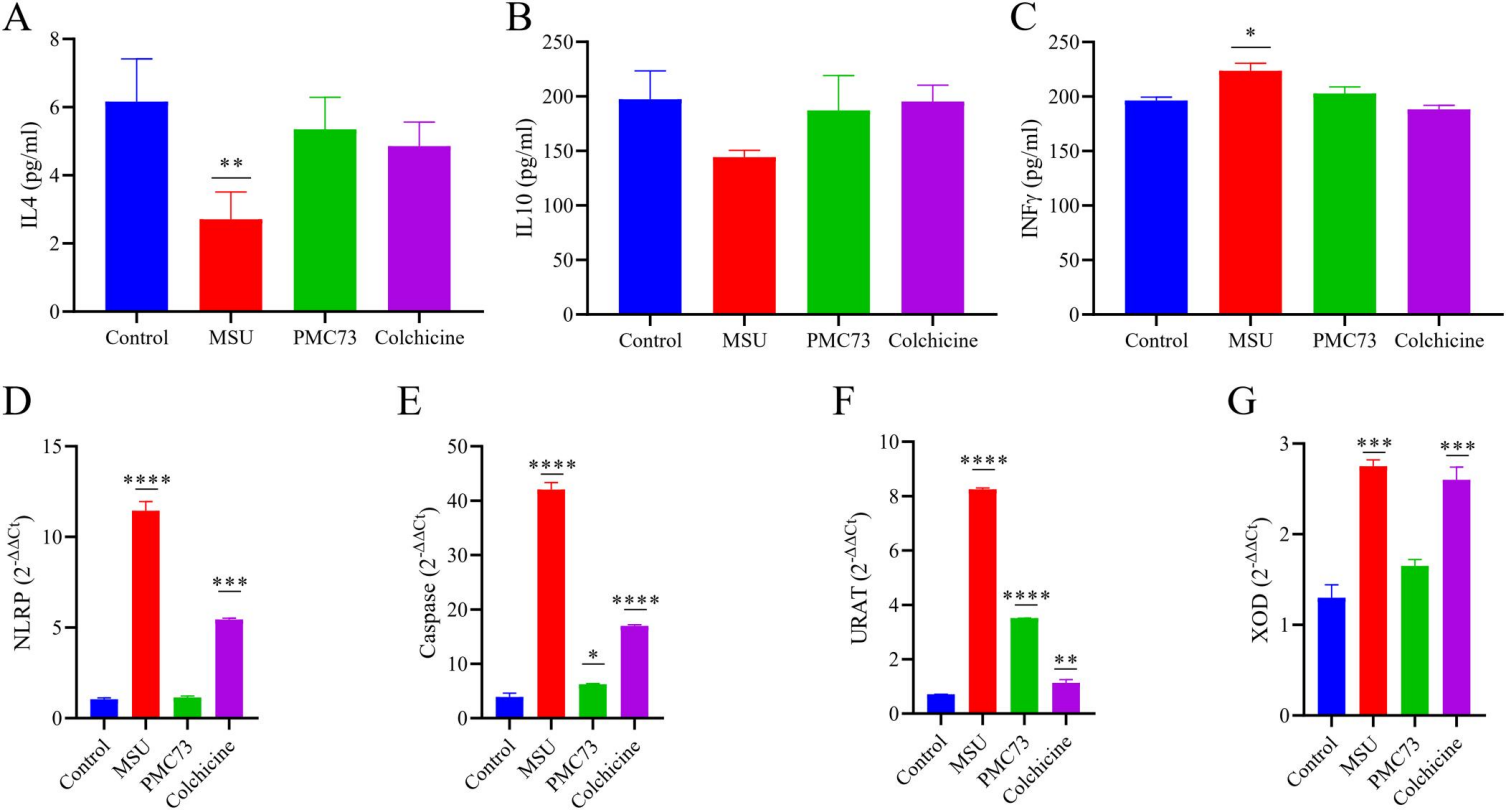
Effects of PMC73 on cytokines and inflammatory gene expression in MSU model. (A–C) Cells supernant cytokine levels (pg/mL) measured by ELISA: (A) IL‐4 decreased by MSU (*p* < 0.01 vs. control) but restored by PMC73 and colchicine; (B) IL‐10; (C) IFN‐γ increased by MSU (*p* < 0.05 vs. control) and normalized by treatments. (D–G) Relative mRNA expression in MSU‐stimulated RAW 264.7 cells: (D) NLRP3, (E) caspase‐1, (F) URAT1, (G) XOD all upregulated by MSU and significantly downregulated (*p* < 0.05, p < 0.01 vs. MSU) by PMC73 and colchicine. Data: mean ± SD (*n* = 3); one‐way ANOVA with post hoc test.

ELISA and RT‐PCR were performed to assess the immunomodulatory effect of PMC73 under inflammatory conditions (Figure 5). As shown in (Figure 5A–C), the MSU‐treated group exhibited a significant reduction in anti‐inflammatory cytokines, particularly IL‐4 (*p* < 0.01), along with a decreasing trend in IL‐10 levels compared to the control group. In contrast, the pro‐inflammatory cytokine IFN‐γ was significantly elevated in the MSU group (*p* < 0.05), indicating an enhanced inflammatory response. Treatment with PMC73 effectively restored cytokine balance. Specifically, PMC73 increased IL‐4 and IL‐10 levels toward normal control values while reducing IFN‐γ production. A similar restorative effect was observed in the colchicine‐treated group. MSU crystal treatment significantly upregulated NLRP3 and caspase‐1 mRNA expression in RAW 264.7 cells compared to NC, indicating NLRP3 inflammasome activation. Treatment with PMC73, colchicine, or their combination markedly suppressed NLRP3 and caspase‐1 expression relative to MSU‐treated controls. PMC73 treatment also downregulated URAT1 and xanthine oxidase (XOD) mRNA expression compared to controls (Figure 5D–G), suggesting reduced uric acid uptake and production. These results collectively indicate that PMC73 attenuates MSU‐induced inflammation by restoring cytokine balance, suppressing NLRP3 inflammasome activation, and reducing uric acid metabolism‐related gene expression.

### 3.6 In Vitro Safety Evaluation and In Vivo Oral Toxicity Assessment

**Figure 6:**
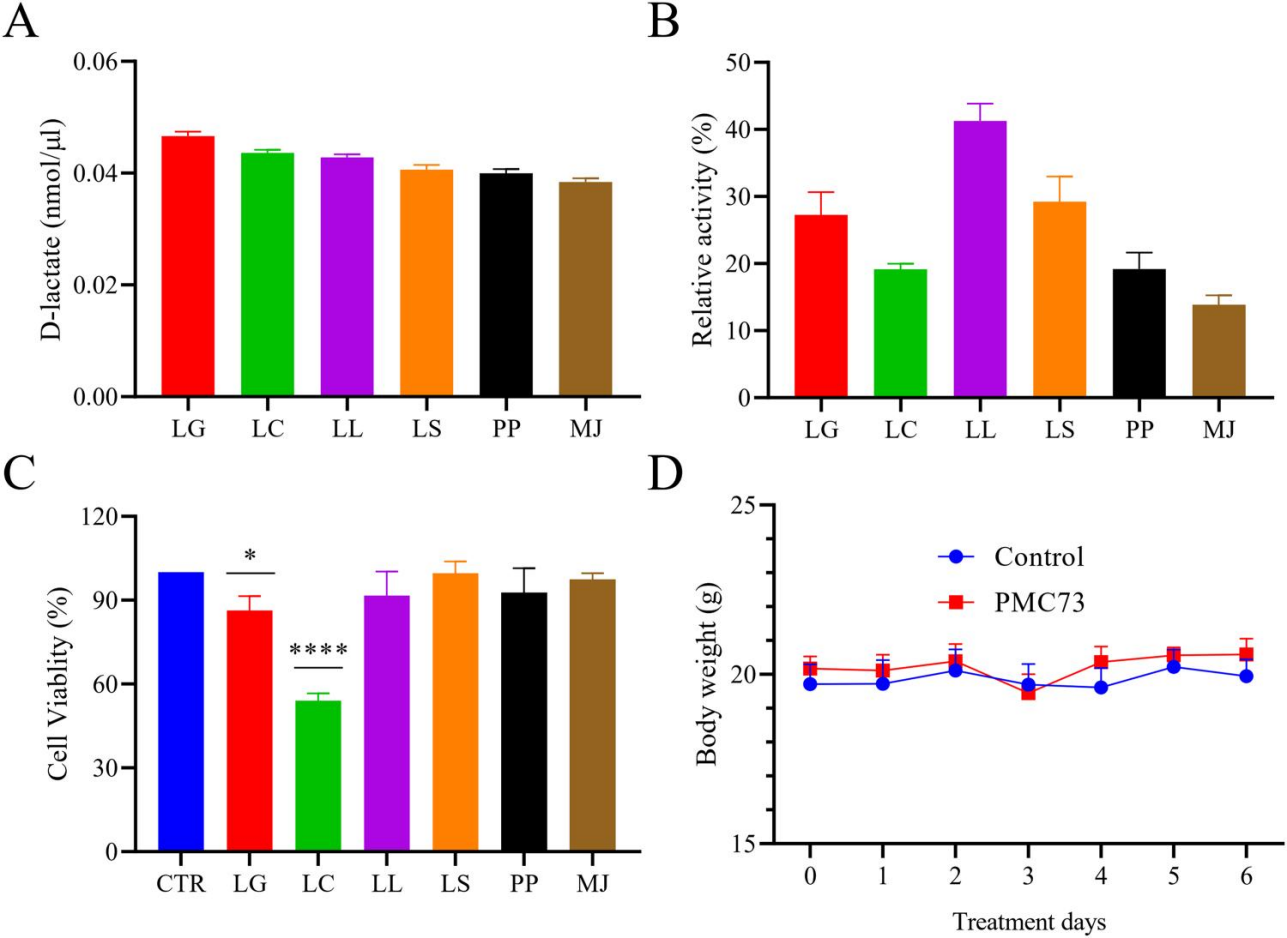
d‐lactate production, antioxidant activity, WST assay and oral toxicity assessment of selected probiotic strains. (A) d‐lactate production (mmol/µL) by LAB extracts; LG and LC exhibited the highest levels, while others showed lower output. (B) DPPH radical scavenging activity (%); LL displayed the strongest antioxidant capacity, with MJ lowest. (C) WST‐1 cell viability (%) after 24 h exposure to probiotics; LC reduced viability to 54.3%, LG to 86.2%, while LL, LS, PP, and MJ remained comparable to Control, viability (*n* = 6). Data represent mean ± SD. (D) Body weight (g) in C57BL/6J mice over 6 days with daily oral PMC73 or saline control; no significant differences (mean ± SEM).

PMC73 and other probiotic strains exhibited favorable in vitro safety profiles and showed no significant toxicity in in vivo oral toxicity assessment (Figure 6). The d‐lactate production results are presented in Figure 6A, with concentrations measured in mmol/µL. Among the tested probiotic strains, *L. graminis, L. curvatus* exhibited measurable d‐lactate levels, while the other strains showed relatively low d‐lactate production. These findings are significant, as excessive d‐lactate production can lead to d‐lactic acidosis, which raises safety concerns in probiotic applications. The antioxidant activity of probiotic strains was determined, and their free radical scavenging activities for DPPH are displayed in Figure 6B. All NGP isolates displayed a moderate to high DPPH scavenging ability of 14% to 42%. Among these isolates, *L. lactis* showed the highest free radical scavenging ability at 42%, compared to ascorbic acid at 99%. In contrast, *M. jalaludinii* showed the lowest scavenging ability at 14%. Cytotoxicity was the primary step in determining the candidate strain's cytotoxic effect on RAW 264.7 cells. The cytotoxicity of candidate strains measured using EZ‐cytox reagent is shown in Figure 6C. In the WST assay, *L. graminis* reduced cell viability to 86.2%, while *L. curvatus* exhibited the strongest cytotoxicity at 54.3% (*p* < 0.0001). *L. lactis, L. sakei, P. pentosacus, and M. jalaudinii* maintained high viability comparable to untreated controls. These results indicate that most extracts are safe at 1 × 10^8^ CFU/mL, though LC requires dose optimization due to consistent cytotoxicity across both assays.

PCR screening of candidate probiotic strain PMC73 for common virulence and antibiotic resistance genes revealed the absence of most tested determinants, indicating a favorable safety profile (Table 4). No amplicons were detected for vancomycin resistance (vanA, vanB), hyaluronidase resistance (hyl), aggregation substance (asa1), or cytolysin (cyl). Similarly, gelE and esp genes were absent (Figure S1). The only positive result was for efaA, which produced an amplicon of the expected size (~ 0.6 kb). The efaA gene encodes an endocarditis‐associated antigen that functions as an adhesin protein, aiding bacterial attachment to host cells. These findings demonstrate that PMC73 lacks key virulence factors associated with pathogenicity in enterococci, supporting its potential as a safe probiotic candidate.

**Table 4:** Screening of virulence gene in candidate strain PMC73.

Category	Gene	Result
Vancomycin resistance gene	*vanA* (Vancomycin resistance)	—
	*vanB* (Vancomycin resistance)	—
Virulence genes	*hyl* (Hyaluronidase)	—
	*asa1* (Aggregation substance)	—
	*ace* (Adhesion of collagen protein)	—
	*gelE* (gelatinase)	—
	*cylA* (Cytolysin)	—
	*esp* (Enterococcal surface protein)	—
	*efaA* (Endocarditis antigen)	+

*Note:* ‘—’, indicate absence and ‘+’ indicate presence of gene.

The PMC73‐treated mice maintained comparable body weights to the saline‐treated control group at all time points measured (days 0–6), with overlapping error bars indicating minimal inter‐individual variability in both groups. No mortality or overt clinical signs of toxicity were recorded in any of the animals during the observation period. No significant changes in body weight were observed in either the PMC73‐treated or control groups throughout the 6‐day treatment period (Figure 6D). As shown in the figure, body weights in both groups remained stable, ranging approximately between 19 and 21 g, with no consistent upward or downward trends detected (Table S1). These findings suggest that oral administration of PMC73 did not induce any measurable toxic effects in male C57BL/6 J mice under the conditions tested.

### 3.7 Gut Microbiota Composition in the HGMS Model

**Figure 7:**
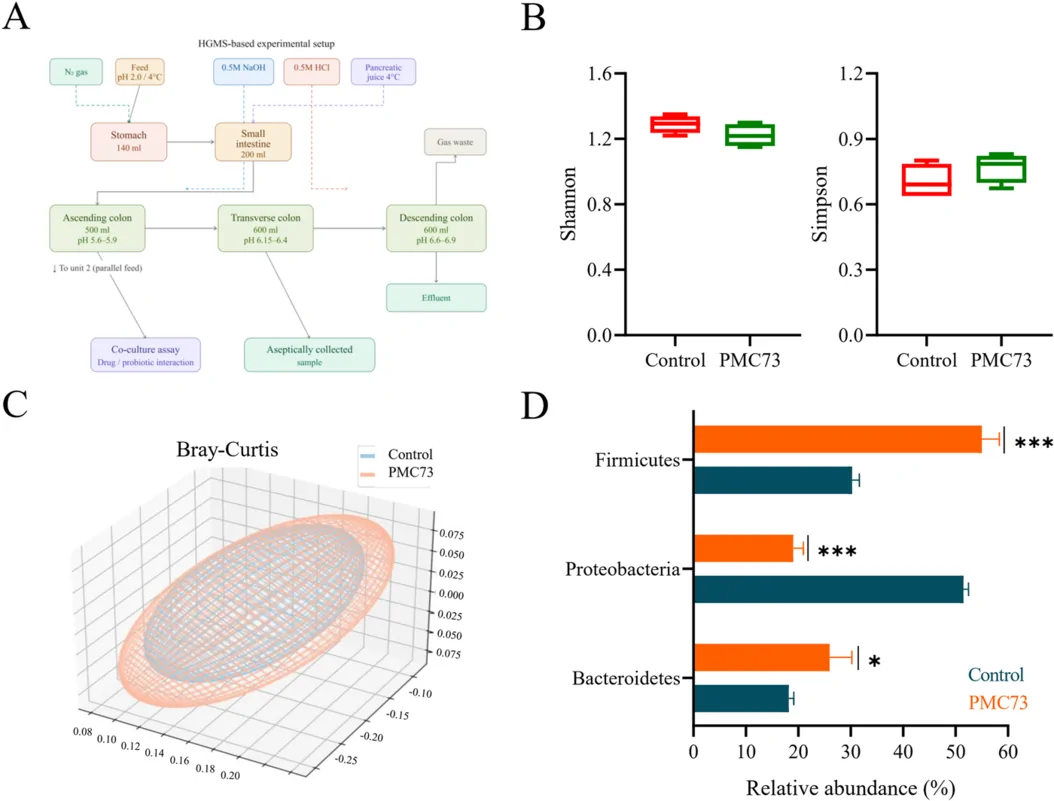
Impact of PMC73 on gut microbiota composition assessed using the ex vivo multi‐treatment Human Gut Microbiota Simulator. (A) Schematic of the MT‐HGMS setup illustrating the flow of feed, pH‐regulated compartments (stomach, small intestine, ascending, transverse, and descending colon), and parallel treatment units. Color‐coded pathways denote material transfer routes. This modified system integrates standard HGMS fermentation modeling with an in vitro co‐culture module, enabling simultaneous evaluation of control, PMC73, and drug–probiotic combinations under controlled anaerobic conditions. (B) Shannon diversity index comparing microbial communities in control versus PMC73‐treated gut samples; no significant differences observed. (C) Bray‐Curtis dissimilarity PCoA plot showing overlap between control and PMC73 communities, indicating similar beta diversity. (D) Relative abundance (%) of major Phylum post‐treatment: Bacteroidetes and Proteobacteria showed modest shifts with PMC73, Firmicutes stable (significance via paired *t* test).

Administration of PMC73 resulted in no significant alterations in alpha diversity, with Shannon index values of approximately 1.25 and 1.20 and Simpson index values of approximately 0.75 and 0.73 in the control and PMC73‐treated groups, respectively (Figure 7B). Beta diversity analysis via Bray‐Curtis PCoA similarly showed substantial community overlap between groups, confirming no significant structural shift in microbial composition (Figure 7C). At the phylum level, PMC73 treatment promoted a favorable compositional shift, with Bacteroidetes increasing from ~18% to ~26% and Firmicutes from ~30% to ~54%. In comparison, Proteobacteria markedly decreased from ~52% to ~19% (Figure 7D). These findings indicate that PMC73 preserves gut microbial stability while selectively enriching health‐associated bacterial populations and reducing opportunistic pathogen‐associated taxa. Overall, PMC73 treatment promoted a more balanced microbial profile, characterized by enrichment of commensal and SCFA‐producing bacteria and reduction of potentially harmful genera.

## 4 Discussion

The present study reports the isolation, characterization, and mechanistic validation of *M. jalaludinii* PMC73, a novel next‐generation probiotic candidate derived from human gut microbiota, with demonstrable anti‐hyperuricemic and anti‐inflammatory efficacy in an in vitro gout model. In this study, we introduce the concept of “Post–Next‐Generation Probiotics (post–NGPs),” defined as microbiome‐derived bacterial strains selected and validated primarily based on their functional bioactivity and mechanistic efficacy rather than their colonization capacity alone (Lin et al. 2019). Unlike conventional NGPs, which emphasize live microbial administration and ecological integration, post–NGPs refer to strains or their biologically active components (e.g., cell‐free supernatants, metabolites, or inactivated cells) that exert reproducible therapeutic effects through defined molecular mechanisms. Regarding the bioactive component underlying PMC73's activity, our experimental design employed different preparations depending on the assay system: whole‐cell suspensions were used for uric acid and purine utilization assays, directly demonstrating intact bacterial metabolic capacity toward uric acid and hypoxanthine degradation, whereas a bacterial extract (8% v/v, derived from 1 × 10^8^ CFU/mL) was used in the MSU‐stimulated macrophage assays to evaluate anti‐inflammatory and uric acid‐lowering effects in a cell‐free, host‐relevant context. This concept aligns partially with the emerging framework of postbiotics but extends it by incorporating strain‐level genomic validation and disease‐targeted functional screening as primary selection criteria. On the contrary, gout remains a significant and growing global health burden, driven by the interplay of dietary, metabolic, and genetic factors that converge on hyperuricemia and MSU crystal‐mediated inflammatory cascades. While established pharmacological interventions provide symptomatic relief and urate‐lowering benefit, their associated toxicity profiles and patient adherence challenges necessitate the exploration of alternative, microbiome‐centered therapeutic strategies.

In humans, purines derived from cellular turnover and dietary intake are catabolized through a stepwise enzymatic pathway: adenine and guanine nucleotides are first degraded to hypoxanthine and xanthine, respectively, and both are subsequently oxidized to uric acid by xanthine oxidoreductase (El Ridi and Tallima 2017)—an enzyme that humans cannot reverse or bypass due to the absence of functional uricase, which was lost during primate evolution. This evolutionary gap means UA accumulates as a metabolic dead‐end, and when renal clearance is insufficient or production is excessive, serum urate surpasses its solubility limit (~ 6.8 mg/dL) (Du et al. 2024), triggering monosodium urate crystal deposition in synovial joints and initiating the NLRP3 inflammasome‐driven inflammatory cascade that defines gout. Hypoxanthine is clinically significant not merely as a precursor but as a flux indicator—its circulating levels reflect the pace of purine turnover and xanthine oxidoreductase activity, and elevated hypoxanthine is increasingly recognized as an early marker of purine overload before overt hyperuricemia is established. Both metabolites therefore serve as mechanistically meaningful targets: UA represents the pathological endpoint, while hypoxanthine represents the upstream bottleneck where interception would preempt UA synthesis altogether. In this study, PMC73 reduced uric acid levels by up to 30% after 48 h of in vitro treatment and grew significantly better in hypoxanthine‐supplemented minimal media (*p* < 0.01), indicating activity at both ends of this metabolic axis. This profile is consistent with documented uricolytic behavior among gut commensals—members of *Lachnospiraceae* (Lou et al. 2024) and *Ruminococcaceae* (Bian et al. 2024) are known to harbor urate oxidase and purinolytic enzymes, and their depletion in dysbiotic microbiomes reliably correlates with elevated serum urate in hyperuricemic cohorts. The hypoxanthine growth advantage further implies the presence of purine nucleoside phosphorylase or xanthine dehydrogenase‐like enzymatic machinery, enabling substrate‐level interception before UA is formed, which inhibit xanthine oxidase at one fixed enzymatic step without engaging the broader purine substrate pool.

The selection of MSU crystal‐stimulated RAW 264.7 macrophages as the in vitro gout model (Dhanasekar et al. 2015) in this study is well‐supported by the literature and represents an established and validated platform for investigating the cellular and molecular mechanisms of gouty inflammation. Macrophages are the principal innate immune effectors in MSU crystal‐induced joint inflammation, serving as the primary cellular sensors of deposited crystals via NLRP3 inflammasome activation (Poulsen and Dalbeth 2025). The significant elevation of uric acid levels following MSU induction (*p* < 0.001) confirmed successful model establishment, consistent with prior reports utilizing this system. The ability of PMC73 to significantly attenuate UA levels within this model, comparable to the positive pharmacological control, validates the functional translatability of PMC73's urate‐lowering activity from cell‐free biochemical assays to a physiologically relevant cellular inflammatory context.

Hyperuricemia is tightly coupled with chronic inflammation, and the NLRP3 inflammasome sits at the center of this relationship (R. Zhang et al. 2018). Once activated by elevated urate, the NLRP3 complex—assembled from its receptor, the ASC adapter, and caspase‐1—drives IL‐1β maturation and release, ultimately fueling renal inflammation and tissue injury (R. Zhang et al. 2018). Beyond inflammation, uric acid homeostasis in the kidney is governed by reabsorption transporters, and dysregulation of these channels is a recognized driver of hyperuricemia. URAT1 and XOD, whose expressions were markedly elevated in MSU‐challenged cells, represent two mechanistically distinct but complementary targets: URAT1 controls intracellular urate reuptake, while XOD catalyzes the terminal step in uric acid biosynthesis (Hoque et al. 2020; Maiuolo et al. 2016). PMC73 treatment significantly attenuated both, suggesting the strain does not merely dampen downstream inflammation but also interferes with the upstream urate‐generating and reabsorption machinery. Colchicine reduced URAT1 to near‐baseline levels but had a comparatively modest effect on XOD, hinting that PMC73 may offer a broader mechanistic reach than the classical anti‐gout reference compound under these in vitro conditions. The changes of XOD level also further confirmed by protein level reduction of XOD using kit method.

Complementing the inflammasome data, cytokine profiling revealed that PMC73 treatment restored immunological equilibrium in MSU‐stimulated macrophages, characterized by significant upregulation of the anti‐inflammatory mediators IL‐4 and IL‐10 alongside suppression of the pro‐inflammatory cytokine IFN‐γ. IL‐10 is a pleiotropic immunoregulatory cytokine produced by macrophages, regulatory T cells, and dendritic cells that exerts broad anti‐inflammatory effects by inhibiting NF‐κB activation, suppressing pro‐inflammatory cytokine transcription, and promoting resolution of acute inflammation (Saraiva et al. 2020). Its upregulation by PMC73 is consistent with immunomodulatory effects attributed to several characterized NGP strains, including *Faecalibacterium prausnitzii*, whose anti‐inflammatory activity has been substantially linked to IL‐10 induction in intestinal macrophages (Sokol et al. 2008). IL‐4, while classically associated with Th2‐mediated adaptive immunity, exerts important anti‐inflammatory functions in macrophages by promoting M2 polarization and suppressing M1‐associated pro‐inflammatory gene expression programs, including those driving IL‐1β and TNF‐α production (Jenkins et al. 2013). The concomitant suppression of IFN‐γ—a potent macrophage activator and amplifier of NLRP3‐dependent inflammation—further reinforces the capacity of PMC73 to shift macrophage functional phenotype from a pro‐inflammatory M1‐like state toward an anti‐inflammatory, resolution‐promoting profile.

The identification of PMC73 as a strain of *M. jalaludinii* PMC73— a species within the family Selenomonadaceae ‐ are strictly anaerobic, Gram‐negative, motile bacteria with fermentative metabolism, typically inhabiting animal gastrointestinal tracts and involved in carbohydrate fermentation and short‐chain fatty acid production (Yeo et al. 2023). The designation of PMC73 as a unique strain through comprehensive genomic and phenotypic characterization distinguishes it from previously described isolates and contributes to the expanding catalogue of human gut‐derived organisms with validated therapeutic functions. Within the broader NGP landscape for hyperuricemia and gout, prior studies have reported urate‐lowering effects for strains including *Lactobacillus plantarum* (Fu et al. 2024) and select *Bifidobacterium* species (Hossain et al. 2025; Vieira et al. 2015); however, these investigations have generally been limited to uricase activity screening or murine in vivo models without the mechanistic depth—encompassing inflammasome biology, transporter regulation, and comprehensive cytokine profiling—achieved in the present study.

A crucial requirement for the clinical translation of any post–NGP candidate is the stringent validation of safety across various standardized parameters. In this regard, PMC73 showed a good and reassuring safety profile across all the tests that were done, such as not producing d‐lactate, not being cytotoxic in cell‐based assays, and not causing gut dysbiosis or oral toxicity in the mouse model. These results are consistent with the safety standards set by the European Food Safety Authority (EFSA), the qualified presumption of safety (QPS) framework, and the Generally Recognized as Safe (GRAS) designation standards of the United States Food and Drug Administration. The absence of d‐lactate production is particularly noteworthy, as d‐lactic acidosis represents a recognized complication associated with certain *Lactobacillus*‐based probiotics in susceptible individuals, including those with short bowel syndrome (Khrais et al. 2022). PMC73 tested negative for the major virulence determinants linked to enterococcal pathogenicity, including vanA/vanB, hyl, asa1, cyl, gelE, and esp, pointing to a low‐risk safety profile. The single positive marker, efaA, is a widely distributed adhesin‐encoding gene rather than a toxin or resistance determinant (Vebø et al. 2010), so its detection alone does not compromise the strain's suitability as a probiotic candidate. Collectively, the safety characterization presented here substantially strengthens the translational credibility of PMC73 and distinguishes this study from prior reports that have proposed probiotic interventions for hyperuricemia without equivalent safety scrutiny.

Despite the promising findings presented in this study, several limitations warrant acknowledgment. First, the anti‐hyperuricemic and anti‐inflammatory efficacy of PMC73 was evaluated exclusively in in vitro systems, namely cell‐free uric acid utilization assays and MSU crystal‐induced RAW 264.7 macrophage models, which, while well‐established and mechanistically informative, do not fully recapitulate the complex physiological, immunological, and microbial ecosystem dynamics of an intact in vivo environment. The extrapolation of these findings to whole‐organism outcomes therefore remains premature and must be validated through rigorous animal model studies prior to any clinical inference. Second, although RAW 264.7 macrophages represent a widely utilized surrogate for innate immune responses in gout research, they are an immortalized murine cell line and may not faithfully reflect the behavior of primary human synovial macrophages or the full cellular repertoire—including neutrophils, mast cells, and dendritic cells—that orchestrates gouty inflammation in human joints. Third, while the downregulation of URAT1 and XOD expression and the suppression of NLRP3‐caspase‐1 signaling were demonstrated at the transcriptional level, the precise molecular mechanisms by which PMC73 or its secreted metabolites mediate these effects remain uncharacterized, and protein‐level validation through Western blotting or proteomics was not performed in the current study. Fourth, The lack of statistical significance in ROS levels between the MSU and PMC73‐treated groups suggests that ROS suppression may not be the primary driver of PMC73's anti‐inflammatory effect in this model, or that the assay's sensitivity was insufficient to detect a modest change; this points toward NLRP3 inflammasome inhibition or downstream cytokine modulation as more likely mechanistic contributors. The conclusions regarding PMC73 and dysbiosis would be further strengthened by providing a more detailed account of the experimental parameters, biological replication, and analytical framework. Fifth, the functional attributes of PMC73 documented here were assessed under standardized laboratory culture conditions, which may not reflect the strain's metabolic activity, viability, or gene expression. Additionally, future studies will include systematic dose‐response comparisons between PMC73 extract and standard positive controls (colchicine, allopurinol) to establish equivalence rationale and determine the optimal therapeutic dosing of PMC73. These limitations are recognized as important directions for future investigation and do not diminish the scientific merit of the present findings but rather define the necessary experimental pathway toward clinical translation.

**Figure 8:**
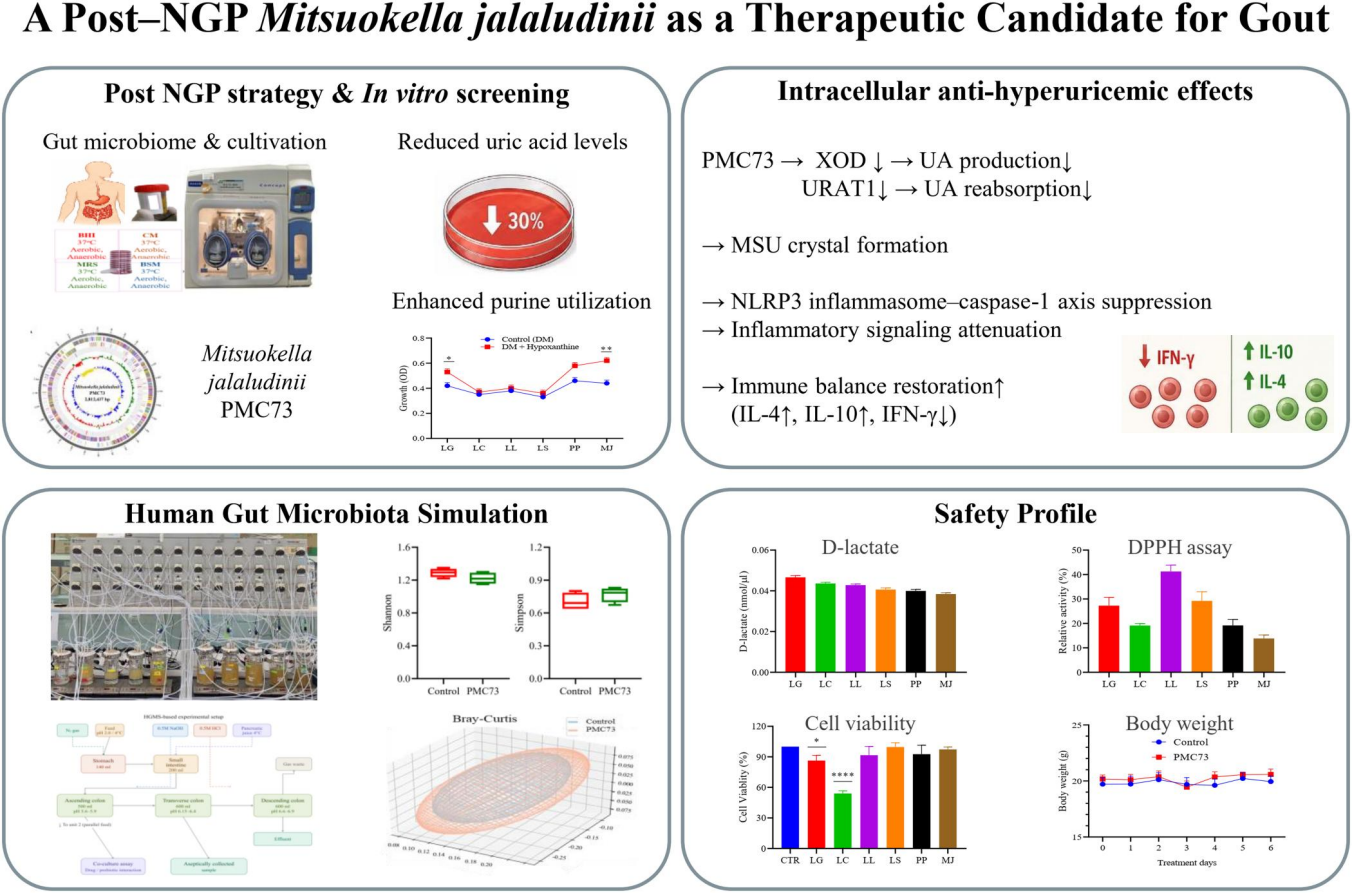
Schematic summary of the therapeutic mechanisms and evaluation of the post–NGP strain *M. jalaludinii* PMC73 for gout. PMC73 demonstrated uric acid–lowering efficacy through purine catabolism and hypoxanthine utilization, along with uric acid reduction in macrophages. Mechanistically, PMC73 suppressed URAT1 and XOD expression, leading to decreased uric acid production and reabsorption. In parallel, PMC73 inhibited NLRP3 inflammasome activation and caspase‐1 expression, resulting in reduced pro‐inflammatory cytokine (IFN‐γ) levels and increased anti‐inflammatory cytokines (IL‐4 and IL‐10). Safety evaluation confirmed a microbiome‐safe profile with no cytotoxicity or in vivo toxicity. These findings support PMC73 as a potential candidate for gout treatment.

In conclusion, this study provides compelling preclinical evidence for the anti‐hyperuricemic and anti‐inflammatory therapeutic potential of PMC73, a novel next‐generation probiotic strain isolated from the human gut microbiome. Through a mechanistically integrated approach, PMC73 was demonstrated to reduce uric acid levels via active purine catabolism, suppress NLRP3 inflammasome activation and caspase‐1 expression, restore pro‐ and anti‐inflammatory cytokine balance, and transcriptionally downregulate both URAT1 and XOD—collectively addressing the metabolic and immunological axes of gout pathophysiology. Its safety profile, established across multiple standardized parameters including cytotoxicity, and murine oral toxicity assessment, further supports its suitability as a candidate for future clinical evaluation. A schematic summary of the proposed mechanisms is presented in Figure 8. Future investigations should prioritize in vivo efficacy assessment in established murine hyperuricemia and gout models, pharmacokinetic and colonization studies to determine optimal dosing regimens, and ultimately, appropriately designed clinical trials to evaluate its safety and efficacy in hyperuricemic and gouty human subjects.

## Author Contributions

**Mohammed Solayman Hossain:** conceptualization, methodology, writing – original draft. **Sukyung Kim:** conceptualization, writing – original draft. **Md Tareque Aziz:** methodology, investigation. **Izaz Ahmaed:** methodology, resources. **Md Sarower Hossen Shuvo:** visualization, formal analysis, software. **Hyewon Yang:** methodology, investigation. **Yunjeong Jang:** methodology, investigation. **Minseo Kim:** methodology, investigation. **Seohyeon Jang:** methodology, investigation. **Yusol Kim:** methodology, investigation. **Soeun Oh:** methodology, validation. **Yunseo Nam:** methodology, investigation. **Hoonhee Seo:** project administration, writing – review and editing, supervision. **Ho‐Yeon Song:** supervision, writing – review and editing, funding acquisition.

## Ethics Statement

This study was conducted following the Declaration of Helsinki. It was approved by the Institutional Review Board (IRB) for Human Research of Soonchunhyang University Seoul Hospital (IRB number: SCH 2019‐12‐004). Written consent was obtained from all participants. For studies involving the Human Gut Microbiome Simulation, approval was obtained from the IRB of Seoul National University Bundang Hospital (IRB No. B‐1810‐497‐308) on 13 October 2023. Animal experiments were conducted under approval (6 May 2025) from the Institutional Animal Care and Use Committee (IACUC) of Soonchunhyang University (Approval No. SCH25‐0028).

## Conflicts of Interest

The authors declare no conflicts of interest.

## Supporting information


Supporting File

## Data Availability

The data that support the findings of this study are available on request from the corresponding author. The genome sequence data for Mitsuokella jalaludinii PMC73 have been deposited in NCBI GenBank under BioProject accession number PRJNA1498720.
