# Supplementary material for: A Post–NGP Mitsuokella jalaludinii as a Therapeutic Candidate for Gout
Source: Microbiologyopen. 2026 Sep 15;15(5):e70410. doi: 10.1002/mbo3.70410 (PMC13576961; doi:10.1002/mbo3.70410)
Supplement: Supplementary file 1 [file MBO3-15-e70410-s001.docx]

Table S1. Individual body weights of Control and PMC73 feed mice

| Body Weight | | | | | | | |
| --- | --- | --- | --- | --- | --- | --- | --- |
| Control | Day 0 (without feeding) | Day 1 | Day 2 | Day 3 | Day 4 | Day 5 | Day 6 |
|  | 18.50 | 22.20 | 20.60 | 20.40 | 20.30 | 19.75 | 20.60 |
|  | 19 | 20.30 | 22.30 | 19 | 18.53 | 19.20 | 19.20 |
|  | 20.10 | 18.95 | 19.65 | 18.40 | 21.50 | 21.90 | 18.40 |
|  | 19.16 | 18.65 | 19.20 | 21 | 19.22 | 20.85 | 21 |
|  | 21.80 | 18.50 | 18.80 | 18.40 | 18.52 | 19.40 | 19 |
| PMC73 | 18.50 | 22.20 | 20.60 | 20.40 | 20.30 | 19.75 | 20.60 |
|  | 19 | 20.30 | 22.30 | 19 | 18.53 | 19.20 | 19.20 |
|  | 20.10 | 18.95 | 19.65 | 18.40 | 21.50 | 21.90 | 18.40 |
|  | 19.16 | 18.65 | 19.20 | 21 | 19.22 | 20.85 | 21 |
|  | 21.80 | 18.50 | 18.80 | 18.40 | 18.52 | 19.40 | 19 |

Figure S1. **Agarose gel electrophoresis analysis of virulence and resistance genes in PMC73 strain.**

PCR amplification was performed using specific primers targeting vancomycin resistance genes (vanA, vanB) and virulence genes (hyl, asa1, ace, gelE, cylA, esp, efaA) with genomic DNA from the PMC73 strain as template, followed by agarose gel electrophoresis. The gel includes an NC (negative control) marker lane and a DNA molecular weight marker lane. Specific bands corresponding to target genes are indicated by brackets, with the prominent band in the efaA lane highlighted.
